# Supplementary material for: Pyruvate Dehydrogenase Kinase 1 inhibition mediated oxidative phosphorylation enhancement in cartilage promotes osteoarthritis progression
Source: BMC Musculoskelet Disord. 2023 Jul 20;24:597. doi: 10.1186/s12891-023-06585-6 (PMC10357736; doi:10.1186/s12891-023-06585-6)
Supplement: Supplementary file 1 — Additional file 1. [file 12891_2023_6585_MOESM1_ESM.zip › MF.Gsea.1653627316242/GOMF_WNT_ACTIVATED_RECEPTOR_ACTIVITY.html]

Details for gene set GOMF\_WNT\_ACTIVATED\_RECEPTOR\_ACTIVITY[GSEA]

|  || Dataset | 55457\_collapsed\_to\_symbols.cls.cls #OA\_versus\_normal.cls.cls #OA\_versus\_normal\_repos |
| Phenotype | cls.cls#OA\_versus\_normal\_repos |
| Upregulated in class | OA |
| GeneSet | GOMF\_WNT\_ACTIVATED\_RECEPTOR\_ACTIVITY |
| Enrichment Score (ES) | 0.4017726 |
| Normalized Enrichment Score (NES) | 1.2224549 |
| Nominal p-value | 0.1958042 |
| FDR q-value | 0.5042221 |
| FWER p-Value | 1.0 |
Table: GSEA Results Summary

  

Fig 1: Enrichment plot: GOMF\_WNT\_ACTIVATED\_RECEPTOR\_ACTIVITY      
 Profile of the Running ES Score & Positions of GeneSet Members on the Rank Ordered List

  

| SYMBOL | TITLE | RANK IN GENE LIST | RANK METRIC SCORE | RUNNING ES | CORE ENRICHMENT || 1 | FZD1 | frizzled class receptor 1 [Source:HGNC Symbol;Acc:HGNC:4038] | 111 | 0.898 | 0.1894 | Yes |
| 2 | FZD7 | frizzled class receptor 7 [Source:HGNC Symbol;Acc:HGNC:4045] | 274 | 0.768 | 0.3462 | Yes |
| 3 | FZD2 | frizzled class receptor 2 [Source:HGNC Symbol;Acc:HGNC:4040] | 1111 | 0.494 | 0.3901 | Yes |
| 4 | ROR1 | receptor tyrosine kinase like orphan receptor 1 [Source:HGNC Symbol;Acc:HGNC:10256] | 1990 | 0.362 | 0.4018 | Yes |
| 5 | TSPAN12 | tetraspanin 12 [Source:HGNC Symbol;Acc:HGNC:21641] | 4214 | 0.136 | 0.2592 | No |
| 6 | LRP5 | LDL receptor related protein 5 [Source:HGNC Symbol;Acc:HGNC:6697] | 4305 | 0.128 | 0.2804 | No |
| 7 | FZD5 | frizzled class receptor 5 [Source:HGNC Symbol;Acc:HGNC:4043] | 5392 | 0.041 | 0.2051 | No |
| 8 | RYK | receptor like tyrosine kinase [Source:HGNC Symbol;Acc:HGNC:10481] | 5896 | 0.002 | 0.1666 | No |
| 9 | FZD8 | frizzled class receptor 8 [Source:HGNC Symbol;Acc:HGNC:4046] | 6084 | -0.011 | 0.1545 | No |
| 10 | FZD9 | frizzled class receptor 9 [Source:HGNC Symbol;Acc:HGNC:4047] | 6812 | -0.064 | 0.1121 | No |
| 11 | FZD4 | frizzled class receptor 4 [Source:HGNC Symbol;Acc:HGNC:4042] | 8159 | -0.168 | 0.0446 | No |
| 12 | FZD10 | frizzled class receptor 10 [Source:HGNC Symbol;Acc:HGNC:4039] | 8354 | -0.182 | 0.0698 | No |
| 13 | FZD6 | frizzled class receptor 6 [Source:HGNC Symbol;Acc:HGNC:4044] | 8379 | -0.184 | 0.1084 | No |
| 14 | PKD1 | "polycystin 1, transient receptor potential channel interacting [Source:HGNC Symbol;Acc:HGNC:9008]" | 8755 | -0.213 | 0.1262 | No |
| 15 | FZD3 | frizzled class receptor 3 [Source:HGNC Symbol;Acc:HGNC:4041] | 9560 | -0.279 | 0.1254 | No |
| 16 | LRP6 | LDL receptor related protein 6 [Source:HGNC Symbol;Acc:HGNC:6698] | 12051 | -0.607 | 0.0659 | No |
Table: GSEA details [plain text format]

  

Fig 2: GOMF\_WNT\_ACTIVATED\_RECEPTOR\_ACTIVITY      
 Blue-Pink O' Gram in the Space of the Analyzed GeneSet

  

Fig 3: GOMF\_WNT\_ACTIVATED\_RECEPTOR\_ACTIVITY: Random ES distribution      
 Gene set null distribution of ES for **GOMF\_WNT\_ACTIVATED\_RECEPTOR\_ACTIVITY**
